# UniRef clusters: a comprehensive and scalable alternative for improving sequence similarity searches

**DOI:** 10.1093/bioinformatics/btu739

**Published:** 2014-11-13

**Authors:** Baris E. Suzek, Yuqi Wang, Hongzhan Huang, Peter B. McGarvey, Cathy H. Wu, the UniProt Consortium

**Affiliations:** ^1^Protein Information Resource, Georgetown University Medical Center, Washington, DC 20007, USA, ^2^Department of Computer Engineering, Muğla Sıtkı Koçman University, Muğla 48000, Turkey, ^3^Center for Bioinformatics and Computational Biology and Protein Information Resource, University of Delaware, Newark, DE 19711, USA, ^4^European Bioinformatics Institute, Wellcome Trust Genome Campus, Hinxton, Cambridge CB10 1SD, UK and ^5^Swiss Institute of Bioinformatics, Centre Medical Universitaire, 1 rue Michel Servet, 1211 Geneva 4, Switzerland

## Abstract

**Motivation**: UniRef databases provide full-scale clustering of UniProtKB sequences and are utilized for a broad range of applications, particularly similarity-based functional annotation. Non-redundancy and intra-cluster homogeneity in UniRef were recently improved by adding a sequence length overlap threshold. Our hypothesis is that these improvements would enhance the speed and sensitivity of similarity searches and improve the consistency of annotation within clusters.

**Results:** Intra-cluster molecular function consistency was examined by analysis of Gene Ontology terms. Results show that UniRef clusters bring together proteins of identical molecular function in more than 97% of the clusters, implying that clusters are useful for annotation and can also be used to detect annotation inconsistencies. To examine coverage in similarity results, BLASTP searches against UniRef50 followed by expansion of the hit lists with cluster members demonstrated advantages compared with searches against UniProtKB sequences; the searches are concise (∼7 times shorter hit list before expansion), faster (∼6 times) and more sensitive in detection of remote similarities (>96% recall at *e*-value <0.0001). Our results support the use of UniRef clusters as a comprehensive and scalable alternative to native sequence databases for similarity searches and reinforces its reliability for use in functional annotation.

**Availability and implementation:** Web access and file download from UniProt website at http://www.uniprot.org/uniref and ftp://ftp.uniprot.org/pub/databases/uniprot/uniref. BLAST searches against UniRef are available at http://www.uniprot.org/blast/

**Contact:** huang@dbi.udel.edu

## 1 Introduction

The UniRef databases (UniProt Reference Clusters) provide clustered sets of sequences from the UniProt Knowledgebase and selected UniParc records to obtain complete coverage of sequence space at several resolutions (100%, 90% and 50% identity) while hiding redundant sequences (Suzek *et al*., 2007). The UniRef100 database combines identical sequences and subfragments from any source organism into a single UniRef entry (i.e. cluster). UniRef90 and UniRef50 are built by clustering UniRef100 sequences at the 90% or 50% sequence identity levels. UniRef entries contain summary cluster and membership information, including the sequence of a representative (best-annotated) protein, member count and common taxonomy of the cluster, the accession numbers of all the merged entries and links to rich functional annotation in UniProtKB to facilitate biological discovery.

The UniRef databases have been produced for 10 years and are used worldwide for a broad range of applications. Since first released in 2004, UniRef has been cited over 400 times based on Google Scholar and unique citations from PubMed Central. UniRef’s ability to reduce redundancy while preserving information on source and quality annotation has proven useful in many studies based on the citation analysis. The most common uses of UniRef databases continue to be in functional annotation, family classification, systems biology, structural genomics, phylogenetic analysis and mass spectrometry. Recent studies have also used UniRef for improving protein sequence alignments through homology extension (Chang *et al*., 2012), increasing sequence search sensitivity with transitive alignments (Malde and Furmanek, 2013), developing representative proteomes and proteome clusters (Chen *et al.*, 2011), predicting the functional effects of disease variants (Capriotti and Altman, 2011a, b; Sim *et al.*, 2012), performing functional screening of metagenomics data (Foerstner *et al.*, 2008; Wommack *et al*., 2012), developing large-scale hierarchical clustering algorithms (Loewenstein *et al.*, 2008), studying gene duplication (Rivera *et al.*, 2010) and conducting genomic studies of peptide and oligonucleotide frequencies (Capone *et al*., 2010). Based on the UniProt usage statistics, UniRef web pages receive approximately 200 000 hits per month. The UniRef file download has been increasing steadily since its inception with an annual growth rate of 20% in recent years, now reaching more than 3000 annual unique IP downloads.

In this article, we present analysis of two additional qualities of UniRef databases: That clusters bring together proteins with similar to identical functional annotation and that similarity searches are faster and as sensitive as searches on native sequence databases. We also provide an update on UniRef database production and coverage.

## 2 System and methods

### 2.1 UniRef database production

The UniRef databases have been produced as a component of UniProt (2013) since its first release in January 5, 2004 and updated with each release of UniProtKB. Production details were previously described (Suzek *et al*., 2007). Briefly, the databases are generated in a hierarchical fashion; UniRef100 clusters are generated first using sequences from UniProtKB and UniParc, UniRef90 clusters are then generated using UniRef100 clusters and UniRef50 clusters are generated using UniRef90 clusters. The clusters are computed using a parallelized version of the CD-HIT algorithm (Li *et al.*, 2001; Li and Godzik, 2006). Using a full update procedure, the clusters are computed *ab initio* at the end of year and are updated for the remaining year using an incremental procedure that favors clustering of new sequences under existing clusters. The representatives of clusters are selected based on the level of curation (reviewed versus unreviewed), protein name (e.g. names do not contain hypothetical or putative preferred), source organism (e.g. proteins from model organisms preferred) and length of protein. The UniRef identifiers are derived from the cluster ‘representatives’ identifiers and are preserved for approximately 98% of the clusters between releases. UniRef production has been continuously enhanced to improve the quality and information content of the databases, as well as the efficiency of cluster computation to cope with explosive growth in sequences being reported.

Starting in January, 2013 an 80% sequence length overlap threshold was introduced for the computation of UniRef90 and UniRef50 databases, that is each member of a given UniRef90 and UniRef50 cluster will have a minimum length overlap of 80% with the longest (seed) sequence. Computed in this manner UniRef is conceptually similar to the PIRSF ‘homeomorphic’ family classification (Wu *et al*., 2004). This overlap threshold prevents proteins sharing only partial sequences from being clustered together. For example, polyproteins and their component proteins, or clusters of domain families partially sharing domain architecture. The threshold also improves intra-cluster molecular function consistency. UniRef100 is computed without the overlap threshold in order to remove sequence redundancy resulting from subfragments. The parallel cluster computation algorithm (Suzek *et al*., 2007) has been revised to accommodate the new overlap threshold.

### 2.2 Characterization of UniRef clusters

To characterize functional properties of UniRef clusters, we assess the intra-cluster molecular function consistency by using Gene Ontology (GO) (Ashburner *et al.*, 2000) molecular function annotations of cluster members in UniProtKB. The consistency between these GO term assignments can be at different levels ranging from all members sharing identical GO terms to members with unrelated GO terms that can only be traced back to the ROOT term in GO hierarchy (i.e. GO:0003674 molecular function). In defining the levels of consistency, we used the GO term specificity (*P*) metric computed based on a term’s hierarchical level:

(1) $$\begin{array}{c} P=1-\frac{\text{Number}\;\text{of}\;\text{Offspring}}{\text{Number}\;\text{of}\;\text{Offspring}\;+\text{Number}\;\text{of}\;\text{Ancestors}} \end{array}$$

(Louie *et al.*, 2010)

where a GO term is more specific (has a larger specificity metric) if the number of ancestors is greater than the number of descendants. The P=1.0 means a GO term has no descendants (the most specific) and P=0.0 means the ROOT term (i.e. GO:0003674 molecular function) that has no ancestors (the least specific).

**Fig. 1.**
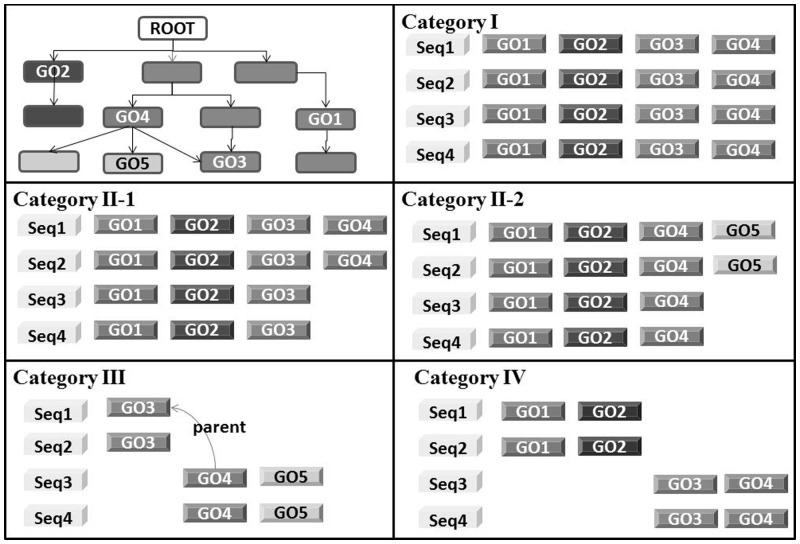
The categories of UniRef clusters based on intra-cluster functional consistency. Upper left panel shows an example of GO term hierarchy used. Other panels illustrate the UniRef clusters in categories based on their intra-cluster consistency; I (All members have identical GO terms), II-1 (all members share common GO terms and some have additional less or equally specific GO terms, not children of the shared GO terms), II-2 (all members share common GO terms and some have additional more specific GO terms), III (only some members share common GO terms but all member’s GO terms can be traced to a common non-root parent GO term, is a child of one of the shared GO terms) and IV (members do not have any common GO term and the existing ones cannot be traced to a common non-root parent GO term)

Accordingly, we categorized UniRef clusters based on their intra-cluster consistency at four levels as shown and described in Figure 1.

### 2.3 UniRef databases for sequence similarity searches

Sequence similarity searches against UniRef database leverage its strength in removing sequencing bias and redundancy, while taking advantage of its power of clustering proteins of similar function (see Section 3.3). Users can avoid compiling and comparing hundreds of BLAST (Altschul *et al*., 1990) result pages containing highly similar, if not identical, sequences. Similarity searches against protein sequence clusters have been shown to be equally sensitive but return faster results (Park *et al.*, 2000; Li *et al*., 2002; Itoh *et al*., 2004; Cameron *et al*., 2007) when compared against native sequence databases of sizes ranging from approximately 400 000 to 2.6 million sequences—several fold smaller than current sequence set used to compute UniRef databases. We tested the effectiveness and performance of UniRef50 for sequence similarity searches as an ever-expanding and continuously updated database available to scientific community.

To do this, we compared results from two full-scale BLASTP searches using UniProtKB/Swiss-Prot query sequences against two separate target databases. UniProtKB/Swiss-Prot is a query set that contains sequences of different length, taxonomy and composition. The first BLASTP search used the UniRef50 cluster seed sequences as a target database and the search results were expanded using the members of the corresponding UniRef50 clusters upon completion of the run (referred to as ‘UniRef50-based searches’). The expanded results included all the subfragments computed at UniRef100 level. The second BLASTP search used all of the UniProtKB sequences as a target database (referred to as ‘UniProtKB-based searches’). In either BLASTP search, the same effective database size (computed based on target consisting of UniProtKB sequences), *e*-value threshold (10) and a large hit list size (10 million) are used as parameters.

The UniRef50-based and UniProtKB-based searches are compared using precision and recall, defined as:

(2) Precision = True PositiveTrue Positive+False Negative= Numbers of hits common to UniProtKB and UniRef50-based searchesNumbers of  hits in expanded  UniRef50-based search

(3) Recall = True PositiveTrue Positive+False Negative=Numbers of hits common  to UniProtKB  and UniRef50-based searchesNumbers of hits in UniProtKB-based search 

where ‘hits in expanded UniRef-50-based search’ consist of all underlying cluster members. These metrics assume the UniProtKB-based searches result in all true hits.

**Fig. 2.**
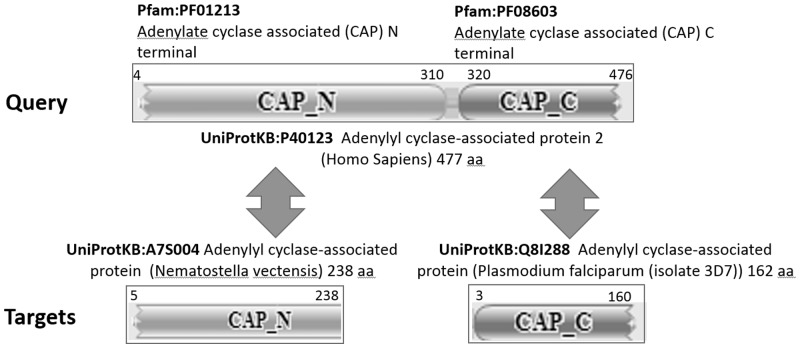
Example UniProtKB/Swiss-Prot (query) and UniProtKB (target) pairs for distant similarity detection analysis, where Pfam domains common to query and targets span more than 80% of target protein sequences

Malde and Furmanek (2013) showed that using UniRef50 as an intermediate database can increase sequence search sensitivity when compared with directly searching the target database. In our study, to compare the ability of UniRef50-based and UniProtKB-based searches to detect distant similarity by BLASTP, we constructed an evaluation dataset based on a different criterion. Pfam domains are detected by curated hidden Markov models, a sensitive means to detect distant similarities that can be expected to find even remotely similar domains. Our dataset contained query–target pairs of UniProtKB/Swiss-Prot Human (query) and UniProtKB (target) protein sequences where query and target share at least one Pfam (Punta *et al*., 2012) domain spanning more than 80% of the target protein. Figure 2 shows an example for targets UniProtKB:A75004 and UniProtKB:Q8I288 that are paired with UniProtKB:P40123 using two domains Pfam:PF01213 and Pfam:PF08603. From a total of 20 247 UniProtKB/Swiss-Prot human sequences, 15 537 paired with at least one target protein meeting our criteria, resulting in 43 523 748 query–target pairs. We also used all InterPro (Hunter *et al*., 2012) domains to construct an alternative evaluation dataset with the same query set and criteria. This dataset contained 831 524 768 pairs for 18 049 queries.

We then counted how many of these query–target pairs are detected by each search. For each human query sequence, we computed the percent difference in related sequences detected by UniRef50-based searches (expanded to all cluster members) versus UniProtKB-based searches. In essence, we try to identify distant similarities missed by UniProtKB- or UniRef50-based searches that are typically identified through domain families.

## 3 Results and discussion

### 3.1 Database coverage, size reduction and cluster distribution

UniRef release 2014_08 (September 3, 2014) was computed from 96 055 068 sequences, including all UniProtKB sequences and isoforms (82 710 662) plus selected UniParc sequences (13 344 406) that were not represented in UniProtKB. It consists of 44 408 603 (UniRef100), 25 890 643 (UniRef90) and 11 862 245 (UniRef50) clusters, with a database size reduction of 54%, 73% and 88%, respectively. Although the sequence space has expanded over 80-fold since the first release in 2004, the parallel CD-HIT clustering algorithm modified for UniRef (Suzek *et al.*, 2007) has proven to be effective in coping with sequence growth. Furthermore, the introduction of the 80% sequence length overlap threshold to the computation of UniRef90 and UniRef50 reduced the compute time more than 5-fold, further improving the scalability of UniRef. Note that the new overlap threshold has little effect on UniRef50 cluster topologies, since it results in less than 5% increase in number of clusters and less than 2% change of representative sequences.

**Fig. 3.**
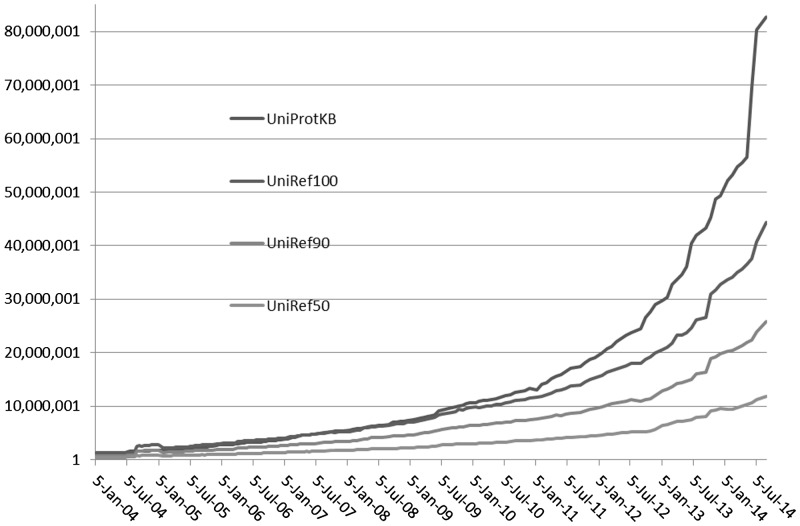
Growth of UniRef databases and UniProt Knowledgebase

The number of protein sequences in UniProtKB continues to rise at an accelerated pace (Fig. 3). Comparing the growth of UniRef with UniProtKB shows a correspondingly improved relative reduction in database size by UniRef, particularly apparent in the last 3 years. The relative reduction in database size went from 5%/42%/70% in 2004 to 54%/73%/88% in 2014 for UniRef100, UniRef90 and UniRef50, respectively, illustrating the effectiveness of UniRef in minimizing sequence redundancy.

**Fig. 4.**
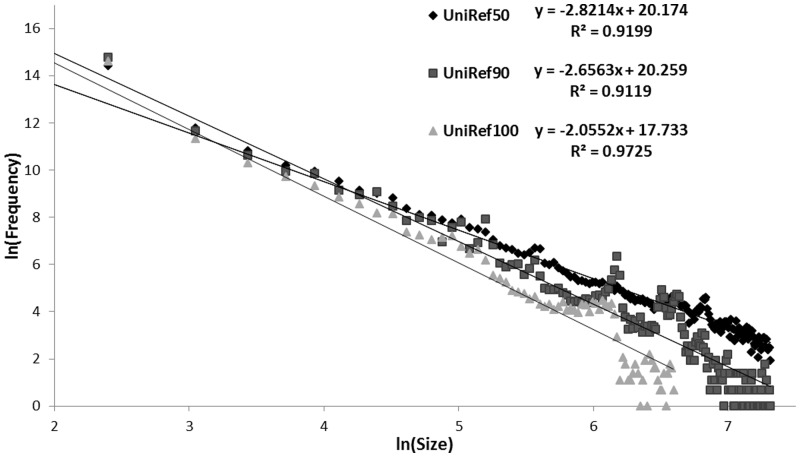
The size distribution of UniRef clusters follows a power law distribution

The size distribution of UniRef clusters continues to follow a logarithmic curve (Fig. 4), consistent with the power law distributions observed in a variety of bioinformatics measures (Luscombe *et al*., 2002; Kunin *et al*., 2005; Lee *et al*., 2005). Single-member clusters constitute a majority of UniRef clusters at all levels; 67% of UniRef50 clusters, 78% of UniRef90 clusters and 89% of UniRef100 clusters have only one member. The largest UniRef100 cluster in the current release 2014_01 contains 9215 highly conserved ‘Histone H3.2’ proteins from eukaryotes, the largest UniRef90 cluster contains 43 060 ‘Ribulose bisphosphate carboxylase large chain’ proteins mainly from eukaryotes and a few unknown organisms (environmental samples), and the largest UniRef50 cluster contains 76 446 ‘Cytochrome c oxidase subunit 1’ proteins from eukaryotes.

### 3.2 Preservation of GO molecular function in UniRef clusters

The evaluation of UniRef clusters and the evaluation of UniRef50-based sequence similarity search (Section 3.3) was conducted using UniRef release 2013_02 (February 6, 2013) following the introduction of the 80% overlap threshold. We used GO molecular function terms for consistency analysis as described in Section 2.2. Only clusters with at least two UniProtKB members annotated with GO molecular function terms, regardless of their evidence codes, were included in the analysis. 1 449 352 UniRef90 clusters and 888 751 UniRef50 clusters met the criteria, account for 50.32% and 41.28% of the multi-member UniRef90 and UniRef50 clusters, respectively. Clusters used in the analysis were categorized based on intra-cluster consistency into four levels (Table 1).

**Table 1.** Categorization of UniRef90/50 clusters based on intra-cluster GO molecular function consistency

Category	Number of UniRef90 cluster (%)	Number of UniRef50 clusters (%)
I. Identical GO terms	1 095 580 (75.59)	586 535 (66.00)
II. Common GO terms	336 821 (23.24)	275 623 (31.01)
III. Common Parent GO terms	13 699 (0.95)	21 113 (2.38)
IV. Inconsistent GO terms	3252 (0.22)	5480 (0.62)
Total number analyzed	1 449 352	888 751

Categories I and II, which represent clusters bringing together proteins of identical and common function, account for 98.83% of UniRef90 and 97.01% of UniRef50 clusters analyzed. In the analyzed set only 0.22% of UniRef90 and 0.62% of UniRef50 clusters were considered inconsistent (Category IV), by the criterion that the lowest shared parent annotated GO term was the ROOT node.

**Fig. 5.**
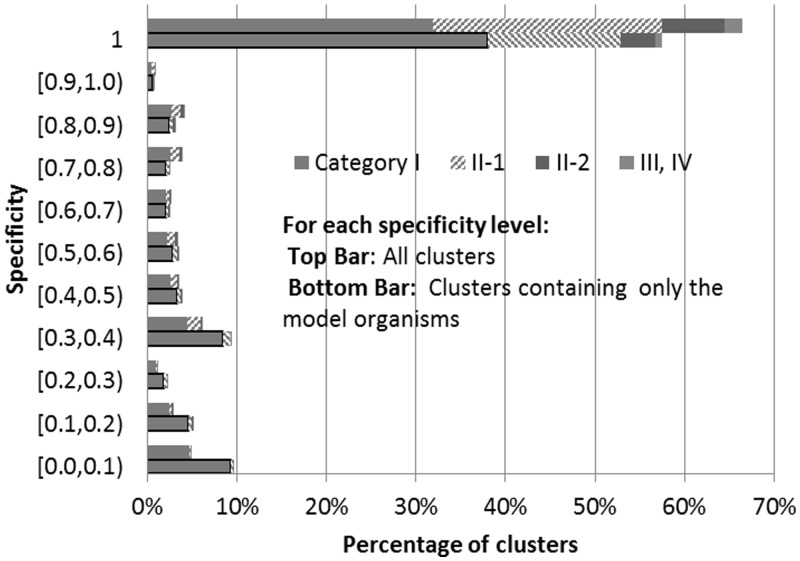
Distribution of UniRef90 clusters specificity for complete set of clusters (top bars) and those containing only model organisms (bottom bars). UniRef50 clusters follow similar distribution

We computed the specificity of a UniRef cluster as the maximum specificity *P* (Equation (1)) for all GO molecular function terms from cluster’s members. Figure 5 shows the distribution of UniRef90 clusters’ specificities where 92% of clusters with specificity 1.0 belong to Categories I and II-1. The common GO terms for these clusters at specificity 1.0 also reach *P* = 1.0. This shows the clusters’ members are consistent on their most specific GO term assignments. At lower cluster specificity levels, Category I still constitutes the majority. Categories III and IV are too small to be compared meaningfully. UniRef50 clusters’ have a category distribution similar to UniRef90.

A more detailed analysis of individual clusters in different categories reveals different functional properties of these categories. Category I clusters constitute the ideal where all members of a cluster are consistently annotated with the same GO terms. For example in release 2013_02, all members of the cluster UniRef90_G1UIH8 are annotated with the same GO terms: ‘GO:0003824 catalytic activity,’ ‘GO:0030170 pyridoxal phosphate binding,’ ‘GO:0008483 transaminase activity’ and ‘GO:0016740 transferase activity.’ The exhibited GO term consistency in annotations, supported with sequence similarity and overlap, could be used to make cluster level GO term assignments.

Category II-1 clusters consist of members annotated with common GO terms and additional GO terms that are no more specific, many of which are parents of common ones. For example in release 2013_02, all the members (when fragments are excluded from UniRef100) of the cluster UniRef50_Q9P225 are annotated with the same GO terms; ‘GO:0005524 ATP binding’, ‘GO:0017111 nucleoside-triphosphatase activity’, ‘GO:0016887 ATPase activity’, ‘GO:0000166 nucleotide binding’ and ‘GO:0003777 microtubule motor activity’. However, only some members of this cluster such as UniProtKB entries P0C6F1 and Q9P225 are also annotated with ‘GO:0003774 motor activity’ that is a parent to ‘GO:0003777 microtubule motor activity’. Such annotation discrepancies are generally corrected in UniProtKB updates. In the case of UniRef50_Q9P225, starting release 2013_10, the members no longer list ‘GO:0003774 motor activity’.

The cases of Category II-2 could be indicative of missing or less specific (under-annotated) GO terms. For example in release 2013_02, cluster UniRef50_I8GP88 consists of ‘d-alanine-poly(phosphoribitol) ligase, subunit 1’ proteins from several Mycobacterium species. All of these proteins are annotated with the corresponding GO term ‘GO:0047473 d-alanine-poly(phosphoribitol) ligase activity’ with the exception of UniProtKB:B1MLB4. This UniProtKB entry is annotated only with the term ‘GO:0003824 catalytic activity’ even if it has the identical sequence (sharing the same UniRef100) with other members of the UniRef50_I8GP88.

Considering GO molecular function annotations in UniProtKB are predominantly assigned using sequence similarity-based methods (e.g. InterPro), we also tested whether our results are biased by a data circularity problem and replicated our consistency analysis on UniRef clusters containing members from 12 model organisms with comprehensive and reliable GO annotation: nine are part of the GO Reference set (Reference Genome Group of the Gene Ontology Consortium, 2009), including *Arabidopsis thaliana*, *Caenorhabditis elegans*, *Danio rerio*, *Drosophila melanogaster*, *Gallus*, *Homo sapiens*, *Mus musculus*, *Rattus norvegicus* and *Saccharomyces cerevisiae*; the remaining three are *Bos Taurus*, *Canis familiaris* and *Sus scrofa*. 55 231 UniRef90 clusters and 44 281 UniRef50 clusters met the criteria. Similar to the analysis on all clusters, in this analysis Categories I and II account for 97.41% of UniRef90 and 94.94% of UniRef50 clusters analyzed. The distribution of clusters at different levels of specificities shared the similar pattern with the analysis conducted using all clusters.

### 3.3 Speed and sensitivity of UniRef50-based sequence similarity searches

To evaluate the speed and sensitivity of UniRef-based sequence similarity searches we compared BLASTP searches using 539 165 UniProtKB/Swiss-Prot entries as query sequences against two target databases—UniRef50 (totaling 6 551 126 seed sequences for all UniRef50 clusters) and UniProtKB (totaling 30 309 136 source sequences used to compute UniRef)—using the same effective database size and identical parameter settings.

For an *e*-value threshold of 10, UniProtKB-based searches returned at least one hit for 535 852 query sequences. Using these results as the ‘gold standard,’ only 183 of them (0.03% of query sequences) do not return any hits in UniRef50-based searches. Among these negative results, 139 are sperm protamine proteins with long stretches of repeating residues (e.g. UniProtKB:P04553), and the remaining are short peptides (<40 residues, e.g. UniProtKB:P84925) or contain repeat or compositionally biased regions (e.g. UniProtKB entries P04368 or P86797) that adversely affect BLASTP searches. On the other hand, 1295 query sequences returned hits on UniRef50-based searches, but not UniProtKB-based searches, all of which are short peptides (<40 residues, e.g. UniProtKB:P83010) except an uncharacterized protein with a poly-asparagine repeat region (UniProtKB:Q54B23).

**Fig. 6.**
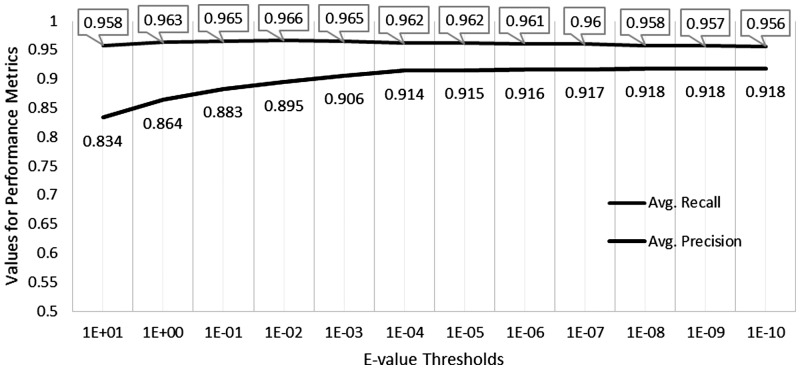
Precision and recall (Equations (2) and (3)) of UniRef50-based BLASTP searches expanded using cluster memberships at different *e*-value thresholds

The average hit list for UniProtKB-based searches is approximately 10 100 entries, while for UniRef50-based searches it is only approximately 1400 entries. When presented in tabular format, the full-scale BLASTP results from UniProtKB-based searches occupy about eight times more space than UniRef50-based searches (450GB versus 38GB). The total computation time is directly proportional to the size of the target database, accordingly UniProtKB-based searches took about 6× more time than UniRef50-based searches (on a 121 core Intel Xeon 2.80 GHz Linux cluster). The precision and recall of UniRef50-based searches, defined assuming all UniProtKB-based search results as correct (Equations (2) and (3)), are shown in Figure 6. The results were evaluated at different *e*-value thresholds ranging 10 to 10e−10 using expanded UniRef50-based BLASTP hits covering all underlying cluster members. The best results plateaued at *e*-value <0.0001, achieving a precision of more than 91% and a recall of more than 96%, respectively.

**Fig. 7.**
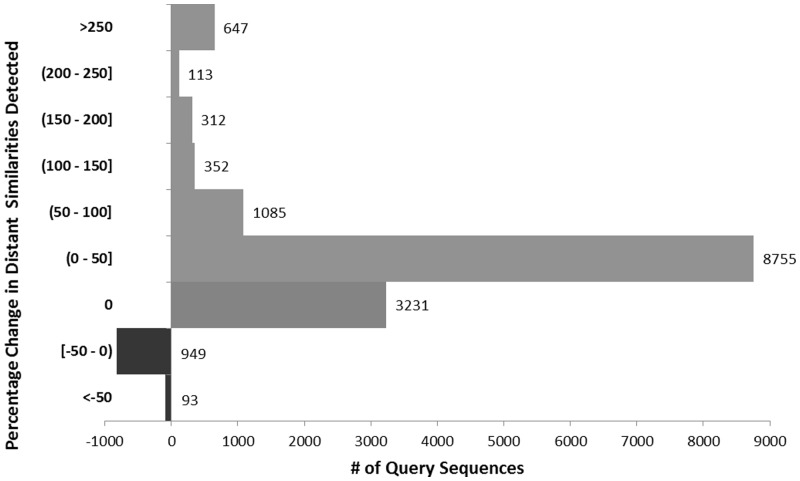
The percentage difference in distant similarities detected by UniRef50- versus UniProtKB-based searches based on the dataset constructed using Pfam domains

To determine how the use of UniRef affects sensitivity in sequence searches, the ability of UniRef50-based searches to detect distant similarity was evaluated using a dataset of query–target pairs constructed using Pfam domains (see Section 2.3). From the total of 43 523 748 query–target pairs, UniRef50-based searches detected a total of 24 602 566 (56.53%) target sequences, whereas UniProtKB-based searches detected 22 955 753(52.74%) targets. Figure 7 illustrates the percentage difference in distant similarities detected by UniRef50-based versus UniProtKB-based searches for 15 537 query sequences where the search detected at least one target with a Pfam domain meeting the 80% overlap criterion. UniRef50-based searches improved detection of distant similarities in 72.50% of the cases at various levels (>0–250%), with a large improvement (>100% increase) in 9.17% of the cases and a moderate improvement (50–100% increase) in 6.98% of cases. On the other hand, in 6.71% of cases (total of 1042), UniProtKB-based searches detected more distant similarities. The InterPro-based analysis has a similar distribution for the percentage differences in detected distant similarities (not shown).

We further examined multiple cases where UniRef50-based searches out-performed or under-performed in comparison with UniProtKB-based searches. Many of the UniRef50 outperforming cases involved clusters where the seed sequence was significantly longer than some of other member sequences. This can happen because there is no overlap restriction on UniRef100 allowing identical and fragment sequences to cluster. For extreme cases where BLASTP queries on UniRef50 found more than 250% more similar proteins than queries on UniProtKB, the majority of the additional sequences found were all marked as fragment sequences. One example in this group is the UniRef50-based search using a 273-aa long query protein ‘Voltage-gated hydrogen channels 1 protein’ (UniProtKB:Q96D96) containing an ‘Ion transport protein’ (Pfam:PF00520), which resulted in the largest percentage increase in detected similarities when compared with UniProtKB-based searches. In this case, the UniProtKB-based search detected only one protein (UniProtKB:C3Z7Z7), a 78-aa fragment sequence, whereas UniRef50-based search detected an additional 217-related proteins from 19 UniRef50 clusters. Among these UniRef clusters, all of their seed sequences were longer than the 273-aa query sequence, ranging from 417-aa to 2745-aa, with 16 seeds being more than 1000-aa and containing multiple copies of the PF00520 domain. In this case all the additional 217 proteins found using UniRef were marked as fragment sequences in UniProtKB ranging in size from 18-aa to 225-aa.

An example where UniRef detected many more remotely similar non-fragment proteins is a query using ‘RNA demethylase ALKBH5’ (UniProtKB:Q6P6C2), a 394-aa human protein involved in RNA demethylation affecting mRNA processing and export and required for spermatogenesis. Q6P6C2 contains a 2OG-Fe(II) oxygenase domain (Pfam:PF13532). The test set contained 2057 query–target pairs. BLASTP against UniProtKB detected only 11 pairs while BLASTP with expansion against UniRef50 detected 821 including all 11 detected using UniProtKB. Only 13 of the additional 810 pairs detected using UniRef50 were marked as fragments. The additional sequences detected by UniRef50 were found in clusters UniRef50_K6KEE3 with 3 members and UniRef50_P05050 with 2312 members. Although BLASTP against UniProtKB did detect a few of the members of UniRef50_P05050 including the seed sequence A6SZ37, it did not detect most of the remotely similar Pfam:PF13532 containing ‘Alpha-ketoglutarate-dependent dioxygenase AlkB’ bacterial proteins involved in DNA repair making up the majority of sequences in this cluster. Alignment of 154 cluster members, 1 for each species contained and ranging in length from 127 to 266 aa, with Clustal Omega (Sievers *et al.*, 2011) showed good alignment throughout the length of the sequences with 28 identical and 27 similar positions distributed throughout the alignment with 9.69% overall identity (data not shown).

In cases where UniProtKB searches out-performed UniRef50, many of the missing proteins had a low degree of similarity to the query and were contained in clusters where the seed sequence was not detected by BLASTP using UniProtKB or UniRef50, effectively masking them from detection. In one example using a 304-aa Human ‘DTW domain-containing protein 1’ (UniProtKB:Q8N5C7) of unknown function containing a DTW domain (Pfam:PF03942, unknown function) as query, we obtained 1589 possible query–target pairs. BLASTP against UniProtKB found 285 of these and against UniRef50 with expansion found 208. There were 102 pairs unique to the UniProtKB results and 8 unique to UniRef50. All of the 102 proteins unique to UniProtKB were 200-aa ‘DTW domain proteins’ from different strains of *Vibrio cholera* and shared a single cluster UniRef50_A7K659 with 270 member sequences. The seed sequence (A7K659) was not detected by BLASTP (<14% sequence identity with Q8N5C7), but 102 slightly more similar cluster members (e.g. D7HA32) were detected using UniProtKB that were hidden when using UniRef50. The eight sequences unique to the UniRef50 search were from four UniRef50 clusters containing members from multiple bacterial species and varying in length from 150 to 208 aa.

**Fig. 8.**
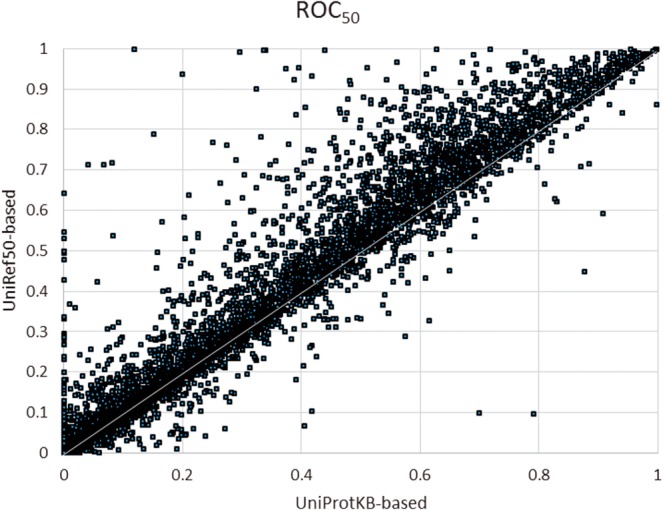
ROC_50_ values for UniRef50- versus UniProtKB-based searches based on the dataset constructed using Pfam domains

We also compared UniRef50- and UniProtKB-based searches using ROC_50_ score (Gribskov and Robinson, 1996), which is the area under the receiver operating characteristic (ROC) curve up to the first 50 false positives. As shown in Figure 8, the ROC_50_ scores for UniRef50-based searches are higher in general when compared with corresponding UniProtKB-based ones (73.4% above diagonal) signifying better sensitivity and specificity.

## 4 Conclusions

In this article, we provide a detailed analysis of intra-cluster molecular function consistency, and assess the suitability of UniRef databases as the target databases in making functional annotation, taking advantage of their low sequence redundancy and bias, especially in the case of UniRef50.

More than 97% of the UniRef90 and UniRef50 clusters were shown to bring together proteins of identical or common molecular function (as captured by GO terms). This outcome reflects the fact that many GO terms are assigned on the basis of sequence similarity-based methods such as InterPro, and further reinforces their use in sequence similarity-based functional annotations. The strong intra-cluster molecular function consistency lends itself to development of new UniRef features such as cluster-level GO annotations whereas the annotation inconsistencies observed in approximately 3% of the clusters is an indicative for a potential use of UniRef databases in detection of annotation errors.

UniRef50-based BLASTP searches are faster (∼6 times), more concise (lists 7 times shorter), and overall more sensitive in detection of remote similarities, while close to complete recall (>96% at *e*-value <0.0001) when compared with UniProtKB-based searches. The hit list consisting of the UniRef50 seed sequences not only reduces the redundancy, but also provides access to information from the corresponding UniRef50 clusters, such as the GO annotations from individual members. In addition, the hit lists when expanded using corresponding UniRef50 cluster members provide an effective way to locate the similar sequences that are already identified by UniRef50 and detect more remote similarities for the query sequence. UniRef50-based searches when compared with UniProtKB-based searches provide the means necessary to identify more easily a shorter list of clusters representing similar proteins from diverse taxa.

In conclusion, our analysis supports the efficiency of using UniRef databases as a powerful alternative to native sequence databases for similarity searches and in using those searches in functional annotation. Our analysis also revealed new uses for UniRef cluster such as correction of GO term annotations through detection of the intra-cluster molecular function incoherencies. UniRef clusters for any UniProtKB entry can be viewed under the ‘similar protein’ section of every entry on the UniProt website.
